# Comparison of body mass index range criteria and their association with cognition, functioning and depression: a cross-sectional study in Mexican older adults

**DOI:** 10.1186/s12877-019-1363-0

**Published:** 2019-12-03

**Authors:** Damaris Francis Estrella-Castillo, Lizzette Gómez-de-Regil

**Affiliations:** 1 Universidad Autónoma de Yucatán. Facultad de Medicina. Licenciatura en Rehabilitación, Avenida Itzáes No. 498 x 59 y 59A. Colonia Centro. Mérida, 97000 Merida, Yucatán Mexico; 2 Hospital Regional de Alta Especialidad de la Península de Yucatán, Calle 7, No. 433 por 20 y 22, Fraccionamiento Altabrisa. Mérida, 97130 Merida, Yucatán Mexico

**Keywords:** Body mass index, Mexican., Elderly., Cognition., Functioning., Depression., Older adults., Ageing.

## Abstract

**Background:**

World population is living longer, demanding adjustments in public health policies. Body mass index (BMI) is widely known and used as a parameter and predictor of health status although an adapted criterion for older adults is usually overlooked. BMI has been extensively analysed in relation to mortality but fewer studies address its association with cognition, functioning and depression in older adults. The present study aimed at 1) comparing BMI distribution according to the ranges proposed by the World Health Organization (WHO) and the United States National Research Council Committee on Diet and Health (CDH), 2) analysing their association with cognitive functioning, physical functioning and depression and 3) analysing a possible, interaction of BMI criteria with sex on the outcome measures.

**Methods:**

This cross-sectional study included 395 participants recruited by convenience sampling; 283 (71.6%) women and 112 (24.58%) men. Mean age was 74.68 (SD = 8.50, range: 60–98). Outcome measures included the Short Portable Mental State Questionnaire for cognitive status, the Barthel’s Index of Activities of Daily Living for physical functioning, and the Geriatric Depression Scale.

****Re**s**ults**:**

WHO criterion classified most cases (65.3%) as overweight, followed by normal weight (32.2%) and underweight (2.5%) whereas CDH criterion considered most (48.1%) as normal weight, and followed by overweight (31.4%) and underweight (20.5%). Analysing cognitive status, independent physical functioning and depression mean scores, significant differences (*p* ≤ .001) were found when comparing the three weight groups (underweight, normal weight and overweight) using either the WHO- or the CDH criterion. Post-hoc tests revealed that in all comparisons the underweight group scored the lowest in all three outcome measures. According to the CDH criterion, overweight was favourable for females but unfavourable for males regarding cognitive status (interaction F(2,389) = 4.52, *p* ≤ .01) and independent functioning (interaction F(2,389) = 3.86, *p* ≤ .05).

**Conclusions:**

BMI and its associations to relevant outcome measures in the older adults must rely on criteria that take into account the particular features of this population, such as the CDH criterion. Underweight was associated with decremented cognition, less independent physical functioning and more depression. Overweight seemed favourable for women but unfavourable for men.

## Background

Population is aging, challenging public policies to respond to the particular health demands of this segment. Accurate body composition information in older adults becomes a relevant aim for research and applied settings, with a potential utility for illness/mortality risk screening, planning, and evaluation of interventions, preventing malnutrition, developing reference standards for ambulatory and non-ambulatory users [1]. Body Mass Index (BMI) is the standard metric of body composition, which adjusts weight-for-height. Although not the only parameter [2, 3], it is certainly the most widely used, probably due to its low cost, simplicity to assess and calculate, and the provision of references by the World Health Organization (WHO) based on international data. The WHO provides cut-off points for adults aged 25 and older (excluding pregnant and breastfeeding women); yet, it acknowledges that in very aged adults BMI is naturally decreased [4].

Some natural physical changes occur even in healthy, successfully aging individuals; for instance: weight loss, sarcopenia (i.e. deficiency of flesh or muscle), increase and redistribution of fat toward the abdomen, loss of bone and body calcium and in consequence, of height [3]. Thus, the WHO criterion for “normal weight” seems less reliable for older adults. From other various criteria, only the one proposed by the United States National Research Council Committee on Diet and Health (CDH) takes into account age stages of adulthood [5].

The prevalence of overweight and obesity is rising, even among older people. A national health survey on Mexican population [6] found overweight and obesity rates of 42.5 and 34.5 in adults aged 60 to 69, 39.0 and 28.3 in adults aged 70 to 79, and 33.8 and 15.7 in adults aged 80 or older [7]. Obesity is associated with higher morbidity and mortality, but in older adults, there is a (debatable) “obesity paradox”. Meta-analyses about the relevance of overweight and obesity to mortality in diverse adult populations suggest that a BMI range of 20–24.9 kg/m2 is optimal for the lowest risk in adults [8, 9]. Yet, when participants with a BMI range of 18.5–20.0 kg/m2 (low, but still normal according to the WHO) was omitted, the beneficial effect of overweight vanished [9]. That led, considering this low BMI group as normal, to the false conclusion that overweight is beneficial.

Beyond the low risk of mortality and morbidity health also implies mental and social well-being. Soon a significant portion of the population will be aged and naturally experiencing health decline. But even while in younger adults higher BMI increases the risk for impaired cognition and late-onset dementia, in late life relates to better cognition [10]. Along with physical and/or cognitive ability decrements care dependence arises up to a point where the individual is no longer able to undertake, without the help of others, the basic daily life tasks [11]. Dependence implies increased health costs and is the main concern and cause of suffering and poor quality of life in older adults [12]. Although in young adults BMI does not seem related to daily life functioning [13], in older adults, it is still controversial. Whereas some studies have found a higher BMI relates to better daily life functioning, even in those with obesity [14], others differ, finding underweight or obese older people subjects to have more limitations than those with normal BMI [15].. Emotional status is also an important aspect of health. Depressive disorders affect about 2–3% of older people living in the community and 10% of those in long-term care facilities. Attention must be given also to sub-threshold depression (i.e. substantial depressive symptoms without meeting the diagnostic criteria), as approximately 1 in 10 older adults is likely to experience it [11]. In older adults, depressive symptoms seem related to both, weight loss and weight gain [16, 17]. Some studies have found no sex differences in this association [16], but others have shown that obesity increases the risk of depression in women, while overweight reduces the risk in men [18].

BMI is widely known and used as a standard parameter and predictor of health status, but adapted criterion for older people are usually overlooked. Few studies have addressed the association of BMI with cognition, functioning, and depression in older adults. The present study aimed at 1) comparing BMI distribution according to the ranges proposed by the WHO and the CDH, 2) analysing their association with cognitive functioning, physical functioning, and depression and 3) analysing a possible, interaction of BMI criteria with sex on the outcome measures.

## Methods

Authorization and ethical approval to perform this cross-sectional study were obtained from the Research and Ethics Committee of the School of Medicine and Rehabilitation of the Autonomous University of Yucatan. Participants were recruited by convenience sampling. Through the study period, three independent senior care centres located in the city of Merida (Mexico), two public and one private, were visited in order to reach users of age 60 or older and invite them to participate. Informed consents were signed voluntarily, granting confidentiality and with no economic compensation involved.

### Measures

Participants were asked to remove their shoes and any garment worn on the head, stand straight, feet together, with head, back, buttocks, calves and heels touching the stadiometer; height was recorded in centimetres. Before every weight measurement the scale was balanced to zero and participants were asked to remove their shoes and any heavy outer clothing. The person should step on the scale platform and stand motionless for a couple of seconds with weight equally distributed on both feet. Weight was recorded on kilograms.

Weight and height were considered to estimate the Quetelet BMI (kg/m^2^) and classify patients according to two criteria. The WHO criterion [19] classifies the status of body composition as: underweight ≤18.49 kg/m^2^, normal ≥18.50 - ≤24.99 kg/m^2^, and overweight ≥25.0 kg/m^2^. The CDH [20] considers weight ranges in people aged 55 to 65 as underweight < 23 kg/m^2^, normal 23–28 kg/m^2^, and overweight > 28 kg/m^2^, and in people aged 66 or older as underweight < 24 kg/m^2^, normal 24–29 kg/m^2^, and overweight > 29 kg/m^2^.

Cognitive status was measured using the Short Portable Mental State Questionnaire (SPMSQ; Pfeiffer questionnaire) [21]. The level of independent physical functioning was assessed with the Barthel’s Index of Activities of Daily Living [22]. Depression was measured with the Geriatric Depression Scale (GDS) [23]. All three questionnaires were applied with their corresponding 10-item Spanish versions. Detailed features of these instruments can be found in a previous report related to this study [24].

### Statistical analysis

Data were collected and analysed with the SPSS v.20 software. First, descriptive statistics (means, standard deviations, frequencies, and percentages) for BMI distribution were obtained and possible differences by sex were explored with t-test and chi-square test. Following, the independent associations of BMI and sex with the three outcome measures were analysed with Pearson correlations and t-tests, respectively. To explore the sensitivity of criteria for the outcome, first a series of one-way analyses of variance (ANOVAs) was run, followed by analyses of covariance (ANCOVAs) adjusting by sex and age. Finally, two-way ANOVAs explored the level of BMI range criteria x sex interaction on the three outcome measures.

## Results

The final sample included 395 participants, 283 (71.6%) women and 112 (24.58%) men. Mean age was 74.68 (SD = 8.50, range: 60–98) and mean BMI was 27.68 (SD = 5.58, range: 17.26–51.61); no significant differences were found for sex. None of the participants reported a health condition that might cause weight loss or gain (e.g. cancer, heart failure, hypothyroidism).

**Fig. 1:**
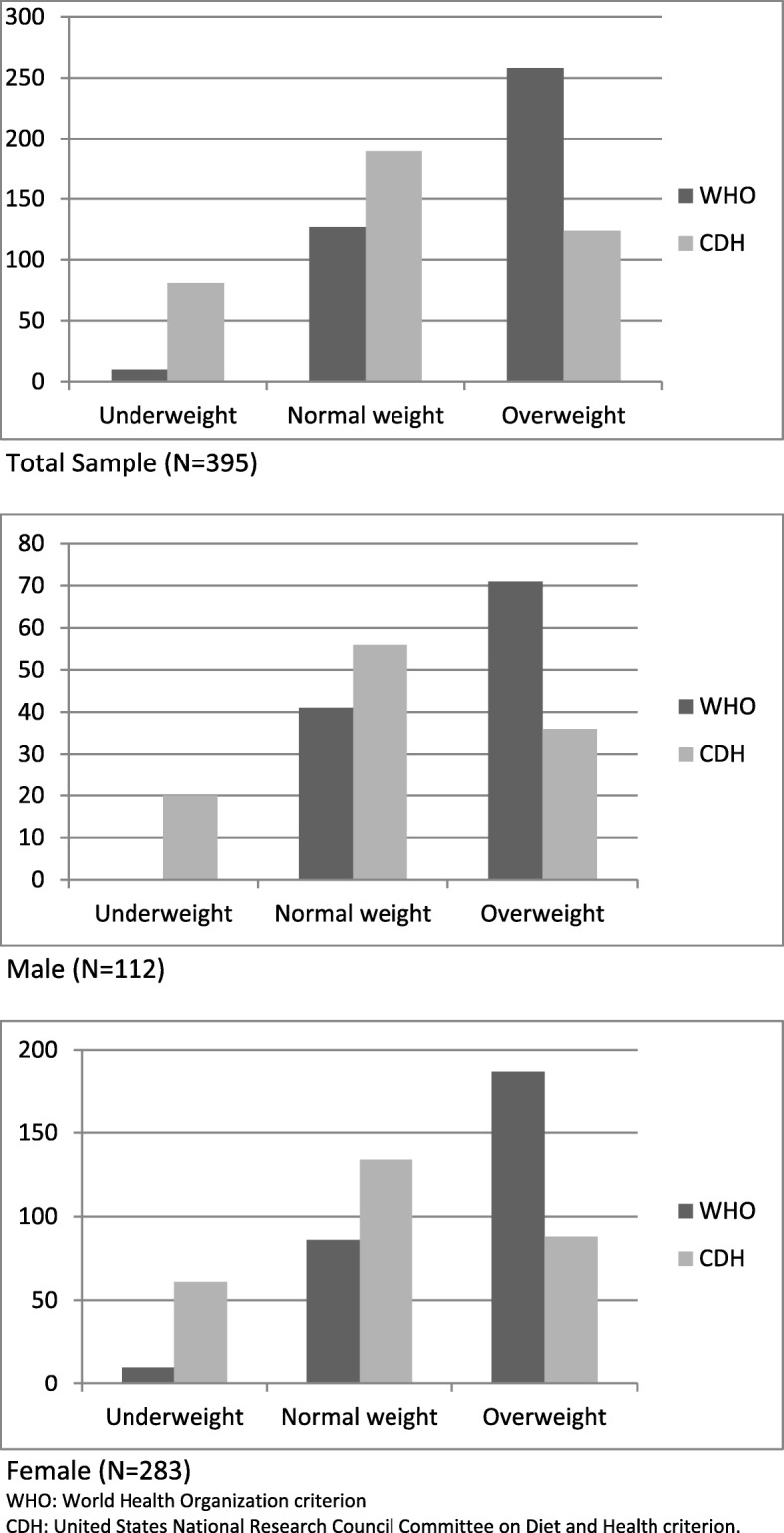
Histograms of BMI distribution according to WHO and CDH criteria

Distribution by BMI according to WHO criterion classifies most cases from total (65.3%) sample and, from the male (63.4%) and female (66.1%) subsamples as overweight. According to CDH criterion, 48.1% from the total sample, 50.0% of male subsample and 47.3% of females subsample have a normal weight. Classification of people as with overweight by WHO criterion seems to have a very low threshold in comparison with CDH criterion, as the WHO percentages approximately double those of CDH (e.g. 65.3% vs. 31.4%). On the other hand, percentages of underweight for total, male and female samples, according to WHO criterion, were 2.5, 0.0 and 3.5, respectively. In contrast, according to CDH criterion, percentages were 20.5, 17.9 and 21.6, respectively. See Table 1 and Fig. 1.

**Table 1:** Sample Body Mass Index Distribution

	Total *N* = 395		Male *n* = 112		Female *n* = 283	
	WHO n (%)	CDH n (%)	WHO n (%)	CDH n (%)	WHO n (%)	CDH n (%)
Underweight	10 (2.5)	81 (20.5)	0 (0)	20 (17.9)	10 (3.5)	61 (21.6)
Normal weight	127 (32.2)	190 (48.1)	41 (36.6)	56 (50.0)	86 (30.4)	134 (47.3)
Overweight	258 (65.3)	124 (31.4)	71 (63.4)	36 (32.1)	187 (66.1)	88 (31.1)

*BMI* Body Mass Index

*WHO* World Health Organization Body Mass Index Criterion

*CDH* United States National Research Council Committee on Diet and Health Body Mass Index Criterion

Significant differences were found when comparing the distribution of participants according to both (WHO and CDH) BMI criteria (χ^2^_(4)_ = 233.20, *p* ≤ .001), as they only coincided in 190 (48.10%) cases. No significant associations between sex and BMI categories, neither according to WHO criterion (χ^2^_(2)_ = 5.01, *p* = .08) nor to CDH criterion (χ^2^_(2)_ = .68, *p* = .71) were found.

Mean outcome scores were: cognitive status 7.31 (SD = 2.56), independent physical functioning 85.28 (SD = 17.37) and depression 2.38 (SD = 1.87). BMI correlated significantly with all three outcome measures; yet, a relevant correlation was only observed with cognitive status (r = +.32, *p* ≤ .001). Men scored significantly higher than women in independent functioning (*p* ≤ .01) and lower in depression (p ≤ .001); they did not score significantly higher in cognitive status (*p* = .50).

Regarding BMI criteria, no extreme values were found for any outcome measure and significant differences were found using either WHO or CDH criterion; yet, the post-hoc tests showed distinct patterns. Regarding cognitive status and depression, WHO criteria suggest that overweight is a favourable factor in older adults, while CDH criteria suggest underweight is disfavorable. WHO criteria coincided with CDH criteria about independent functioning; underweight older adults had a poorer performance in comparison to normal- and overweight older adults, who showed no significant differences between them. CDH criteria found a poorer condition of older people with underweight as they have lower cognitive and independent functioning and more depression; older people with overweight were in better conditions than older people with normal weight, though differences were not significant. WHO criteria found a better functioning in older people with overweight; particularly regarding cognitive status and depression. The associations of BMI (WHO and CDH criteria) with the three outcome measures remained significant even after adjusting by sex. When adjusting by age, the associations were still significant for cognitive status and independent physical functioning, but not for depression. Table 2 summarizes the results.

**Table 2:** Differences in Cognitive Status, Independent Physical Functioning and Depression Scores According to WHO- and CDH Body Mass Index Criteria

	Cognitive status Mean (SD)		Independent physical functioning Mean (SD)		Depression Mean (SD)	
	WHO	CDH	WHO	CDH	WHO	CDH
Underweight (U)	4.50 (2.84)	5.72 (2.77)	55.00 (15.99)	75.99 (20.86)	3.90 (2.28)	3.13 (2.34)
Normal weight (N)	6.50 (2.85)	7.62 (2.39)	83.43 (18.73)	86.63 (16.37)	2.74 (2.10)	2.24 (1.69)
Overweight (O)	7.81 (2.22)	7.86 (2.28)	87.36 (15.48)	89.27 (13.98)	2.14 (1.68)	2.10 (1.68)
F_(2,392)_	18.96***	21.69***	19.45***	16.68***	8.04***	8.63***
Power	1.000	1.0000	1.000	0.999	0.909	0.970
Post-hoc	U < N* U < O*** N < O***	U < N*** U < O*** N < O	U < N*** U < O*** N < O	U < N*** U < O*** N < O	U > N U > O** N > O**	U > N*** U > O*** N > O
Controlling by Sex						
F_(2,392)_	18.85***	21.49***	18.24***	16.37***	7.88***	8.34***
Controlling by Age						
F_(2,392)_	7.70***	10.09***	9.39***	5.26**	1.75	2.26

BMI: Body Mass Index

*WHO* World Health Organization Body Mass Index Criterion

*CDH* United States National Research Council Committee on Diet and Health Body Mass Index Criterion

*p ≤ .05, **p ≤ .01, ***p ≤ .001

**Fig. 2:**
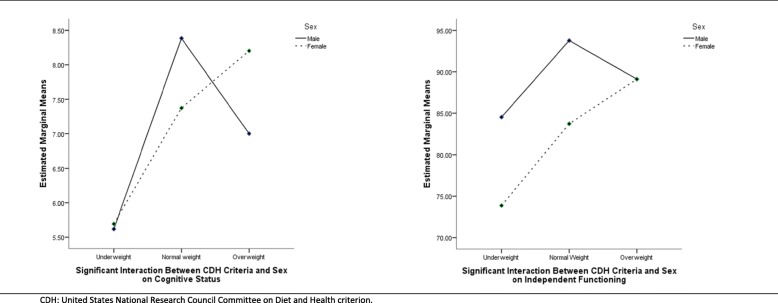
Graphic distribution of the Significant Interactions of CDH criteria with Sex on Cognitive Status and Independent Functioning

The interaction of WHO BMI criteria with sex was not significant for any of the studied outcome measures. Yet, CDH BMI criteria seemed more sensible to differences as its interaction with sex found significant results for cognitive status (F_(2,389)_ = 4.52, *p* ≤ .01, η^2^ = .023, power = 0.769) and independent functioning (F_(2,389)_ = 3.86, *p* ≤ .05, η^2^ = .019, power = 0.698). Underweight is disfavorable for cognitive status and independent functioning, particularly in women (Fig. 2). There is a trend for improvement as BMI goes from underweight to normal weight. Yet, regarding overweight, this seems favourable in women but not in men. No significant interactions were found for depression.

## Discussion

This study aimed at comparing, in a sample of Mexican older people, BMI distribution according to two alternative ranges as proposed by the WHO and the CDH. Results showed that having the WHO criteria a lower threshold for what is to be considered a normal weight in comparison to the CDH criteria; it classified more than half the participants as overweight, that is, the double if considering the CDH criteria. Furthermore, the CDH criteria displayed a distribution wherein half of the sample had a normal weight range. These differences in distribution patterns, in concurrence with previous research [5], bring into question the adequacy of the common practice of using the WHO criteria to classify normal BMI in older adults. The accuracy of BMI seems to diminish with age, as body composition changes increase adiposity and sarcopenia (i.e. decreased muscle mass) [25]. Valid, reliable and economical assessments of BMI, with ranges adapted by age, are needed [5, 26].

Analyses of the association of BMI criteria with cognitive functioning, physical functioning and depression revealed similar though not equivalent patterns. Considering the WHO BMI criteria, overweight seems a protective factor. Results from some studies, mostly relying on the WHO BMI criteria, suggest that overweight is a protective rather than a risk factor (at least for mortality) in older people [27]. Yet, it has been questioned whether this BMI paradox just reflects the WHO criteria’s low sensitivity for this segment of the population [25, 26]. Classifying sample by the CDH BMI criteria, adapted by age range, significant results point to the opposite direction; that is, underweight is disfavorable in older people. This concurs with studies reporting low BMI to be disfavorable in older people, and highly associated with infections, hospitalizations and predicting mortality [28, 29]. Also, a rapid and unintentional weight loss may reflect underlying illness, social deprivation, dementia or depression [28].

After the age of 60, average body weight and muscle mass tend to decrease. As physical activity and energy expenditure also decrease there is a tendency to fat accumulation and fat redistribution [30]. Here, underweight older people showed a disadvantageous performance, while in other studies overweight older people showed a more favourable status [27]. However, one must be cautious; obesity in older people is a common and serious matter of concern not to be overlooked. Not only can obesity lead to adverse health consequences and impair quality of life, but also exacerbate the age-related decline in physical function and lead to frailty, disability and autonomy limitations [29, 31–33]. Treatment for obesity in older persons is controversial, mainly to the misinterpretation that it may not be as harmful in older adults as it is in younger people, and the concern about the potential adverse effects of weight loss in this population [28, 31, 33–35]. Even small amounts of voluntary weight loss (between 5 and 10% of initial body weight) along with a healthy lifestyle may benefit older people [32]. Weight loss in overweight/obese older people can improve risk factors, fat loss can ameliorate certain catabolic conditions of aging through impacting muscle protein synthesis and breakdown and lighter weight may also ease the mechanical burden on weak joints and muscle, thus improving mobility [28]. Interventions aiming at voluntary weight-loss in obese older people must follow a combination of exercise and modest calorie restriction for reducing intra-abdominal fat mass while muscle mass and strength are preserved [30, 31, 33, 35]. Moreover, interventions must consider comorbidities, polypharmacy, limitation of autonomy, and social issues with a focus on the underlying medical problems, functional status and living environments [34].

Cognitive status, independent physical functioning, and depression are three important outcome measures in older adults that have been found related to BMI. Although some studies have found a poorer cognitive performance in the overweight and obese older people [36] our results concur with those finding lower BMI coinciding with a worse cognitive status [37]. Regarding physical functioning, studies tend to support that high BMI values are associated with greater functional impediments [38]; yet, it has also been found that both, low and high BMI are related to a greater risk of functional impairment [39]. The present results found poorer physical functioning in underweight older people following the CDH criteria. Depressive symptoms in older adults seem less likely to occur in overweight/obese older people [40], and that is the case in our study if the WHO criterion is used. If the CDH criterion is used, underweight older people seem more likely to report depressive symptoms, and that coincides with previous findings, particularly in men [40]. Discrepancies in findings might be due to the use of diverse measures for body composition, cognition, functioning, and depressive symptoms, and the fact that these outcome measures have not been previously studied together.

Regardless of BMI criteria, the group of underweight older people had a disadvantageous outcome on all three measures in comparison to the other groups. Furthermore, results showed that considering its interaction with sex, underweight is disadvantageous for all, whereas overweight is favourable in women but disfavorable in men. These results evidence that a criterion overlooking age and sex differences in BMI may bias research findings and perhaps explain the so-called obesity paradox in older adults. More complex models including covariates that might influence outcome, such as educational level, regular cognitive stimulation, comorbidities, medication intake and mental health history should be considered to support or disclaim these results.

Besides, underweight women stand out as the more vulnerable group regarding cognitive status and independent physical functioning. Nutritional interventions must aim at helping older people to gain weight up to normal status (rather than reaching overweight) but considering a more flexible cut-off point such as suggested by the CDH. That is, a healthy BMI in older people must range between 23 and 28 in people aged 55 to 65, and between 24 and 29 in people aged 66 or older. Furthermore, priorities for intervention should be given to those at highest risk, with the primary focus on reducing the risk profile rather than weight loss per se [4].

It must be underscored that more weight does not equal better nutrition or good health. Given the varying contributions of bone mass, muscle mass and fluid to body weight, relying exclusively on BMI to classify individuals may result in misclassification. Anthropometric data for the potential development of reference data or standards should cover at least weight and height, plus age, sex, race, socioeconomic status, presence of disease, and smoking habits [4]. Relying on convenience sampling limits the generalization of results as the selected group may not be comparable to others, such as older adults healthy and living independently. Moreover, when studying BMI in older adults it would be worth exploring possible differences due to receiving care from others, either at home or in care centres, and observing its evolution through time. As sex and age were recorded, their role as possible confounders was analysed; although the significance of most results was confirmed, only when adjusting by age, the association was no longer significant for depression. Further research must also consider the inclusion of other possible confounders such as disease status, smoking status, alcohol intake, physical activity, socioeconomic status and education for a better understanding of the processes regulating the associations of BMI with outcome.

Despite its limitations, this study showed that when assessing BMI in older people, a criterion adapted by age must be preferred. It seems that the WHO criteria overshadow a problem in the older population, namely that losing weight is in fact unfavorable, leading to a lower BMI. The CDH criteria are much more sensitive to that problem. Furthermore, the fact that WHO cut-off points are more restrictive may help explaining why various studies using this criterion found overweight to be favourable in older people. The use of CDH cut-off points showed that overweight is not a protective, neither a risk factor in older people, at least in relation to our 3 outcome measures. In older people, underweight is what signals a high risk of mortality, and in line, this study shows also a higher risk of cognitive and functional deterioration. Interventions for weight control in older people must monitor healthy weight gain but prevent obesity.

## Conclusions

In this study, the CDH seemed a more sensitive BMI criterion than the WHO’s and could be recommended. CDH criteria not only showed a more sensible distribution in BMI but also found significant differences in the selected outcome measures and some significant interactions with sex. Underweight in older adults was related to decremented cognition, less independent physical functioning, and more depression. Overweight seemed favourable for women but disfavorable for men.

## Data Availability

The datasets used and/or analysed during the current study are available from the corresponding author on reasonable request.

## Abbreviations

ANOVA: Analysis of variance
BMI: Body Mass Index
CDH: The United States National Research Council Committee on Diet and Health
GDS: Geriatric Depression Scale
SPMSQ: Short Portable Mental State Questionnaire
WHO: World Health Organization
